# Silicone-layered waterproof electrohydraulic soft actuators for bio-inspired underwater robots

**DOI:** 10.3389/frobt.2024.1298624

**Published:** 2024-06-14

**Authors:** Takumi Shibuya, Shuya Watanabe, Jun Shintake

**Affiliations:** Department of Mechanical and Intelligent Systems Engineering, School of Informatics and Engineering, The University of Electro-Communications, Tokyo, Japan

**Keywords:** bio-inspired robotics, underwater robots, electrohydraulic soft actuators, HASEL actuators, soft robotics

## Abstract

Electrohydraulic soft actuators are a promising soft actuation technology for constructing bio-inspired underwater robots owing to the features of this technology such as large deformations and forces, fast responses, and high electromechanical efficiencies. However, this actuation technology requires high voltages, thereby limiting the use of these actuators in water and hindering the development of underwater robots. This paper describes a method for creating bio-inspired underwater robots using silicone-layered electrohydraulic soft actuators. The silicone layer functions as an insulator, enabling the application of high voltages underwater. Moreover, bending and linear actuation can be achieved by applying the silicone layers on one or both sides of the actuator. As a proof of concept, bending and linear actuators with planar dimensions of 20 mm × 40 mm (length × width) are fabricated and characterized. Underwater actuation is observed in both types of actuators. The bending actuators exhibit a bending angle and blocked force of 39.0° and 9.6 mN, respectively, at an applied voltage of 10 kV. Further, the linear actuators show a contraction strain and blocked force of 6.6% and 956.1 mN, respectively, at an applied voltage of 10 kV. These actuators are tested at a depth near the surface of water. This ensured that they can operate at least at that depth. The actuators are subsequently used to implement various soft robotic devices such as a ray robot, a fish robot, a water-surface sliding robot, and a gripper. All of the robots exhibit movements as expected; up to 31.2 mm/s (0.91 body length/s) of locomotion speed is achieved by the swimming robots and a retrieve and place task is performed by the gripper. The results obtained in this study indicate the successful implementation of the actuator concept and its high potential for constructing bio-inspired underwater robots and soft robotics applications.

## 1 Introduction

Bio-inspired underwater robots are being actively studied owing to their potential for achieving high maneuverability and efficiency due to their ability to mimic and draw inspiration from the structures and movements of underwater organisms. These robots have the potential to aid in tasks such as exploration, monitoring, and rescue missions and contribute to the understanding of the biomechanics of aquatic animals. The use of soft actuators is a promising approach for constructing bio-inspired underwater robots (Aracri et al., 2021; Jian and Zou, 2022; Wang R. et al., 2023; Li et al., 2023) because robots with biological and simple structures can be realized. Researchers have proposed several soft underwater robots based on various soft actuators made of materials such as ionic polymer-metal composites (Takagi et al., 2006; Aureli et al., 2010), lead zirconate titanate (Shintake et al., 2010; Xing et al., 2023), shape memory alloys (Villanueva et al., 2011; Coral et al., 2018), fluidic elastomer actuators (Katzschmann et al., 2018; Nguyen and Ho, 2022), and dielectric elastomer actuators (Shintake et al., 2018; Li et al., 2021).

Among the different soft-actuation technologies that presently exist, electrohydraulic soft actuators are promising for constructing bio-inspired underwater robots (Rothemund et al., 2021). These actuators can generate large deformations and forces or pressures (79% and 25 kPa, respectively) with relatively high frequencies of up to 20 Hz and an electrical-mechanical efficiency of 21% (Acome et al., 2018; Mitchell et al., 2019). Despite these promising features, only a jellyfish robot has been reported as an example of an underwater robot using electrohydraulic soft actuators (Wang T. et al., 2023). This may be because actuator technology is relatively new and requires high voltages, typically a few kilovolts. This indicates that further research on electrohydraulic soft actuator-based underwater robots, including establishing fabrication methods and investigating robot geometries and swimming modes, is an important initiative to advance their use in underwater systems.

**FIGURE 1:**
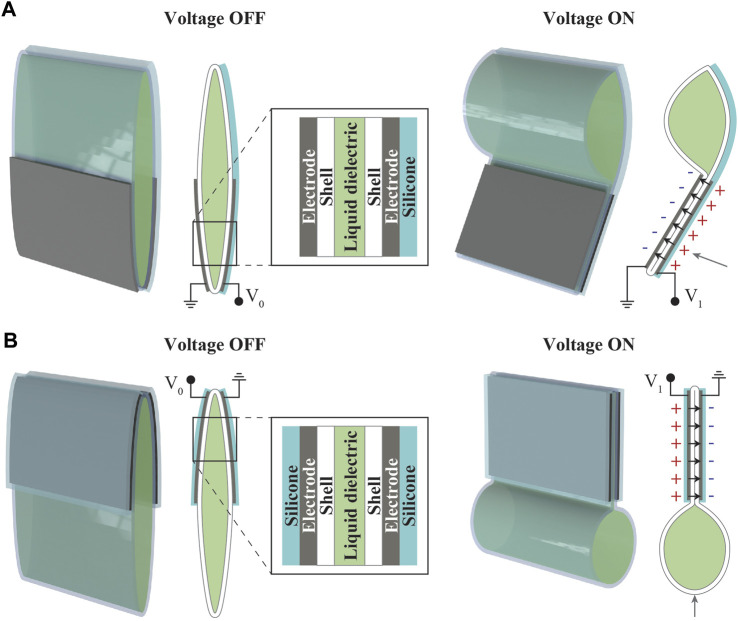
Structure and working principle of silicone-layered electrohydraulic soft actuators. **(A)** Bending and **(B)** linear actuators.

In this study, we describe a method to construct bio-inspired underwater robots based on silicone-layered electrohydraulic soft actuators. The actuators consisted of a flexible shell filled with liquid dielectric and electrodes on both sides. As shown in Figure 1, these actuators are further equipped with one or two highly compliant silicone layers that function as an insulator, enabling the application of high voltages underwater. When voltage was applied, electrostatic forces were generated between the electrodes, causing the electrodes to attract each other. The movement of the electrodes and flexible-shell caused the liquid dielectric inside to move, resulting in actuation. Here, the presence of a silicone layer also determined the actuation behavior. When the silicone layer was placed on one side of the actuator, a bending deformation was generated due to asymmetric flexural rigidity (Figure 1A). When the actuator was equipped with two silicone layers on both sides, a linear deformation was generated (Figure 1B). The actuators were characterized and applied to construct various underwater devices, including ray and fish robots. These robots exhibited locomotion in water with different swimming modes such as flapping and oscillation, demonstrating the versatility of our method for developing electrohydraulic soft actuators for bio-inspired underwater robots.

## 2 Materials and methods

### 2.1 Materials

A 20 µm heat-sealable oriented polypropylene (OPP) film (P3162, Toyobo) was used as the flexible shell of the actuator. A conductive double-sided tape (CN4490, 3M) was used to form the electrodes, an insulating oil (FR3, Cargill) was used as the liquid dielectric, and Ecoflex 00-30 (Smooth-On) was used as the silicone layer.

### 2.2 Fabrication process

**FIGURE 2:**
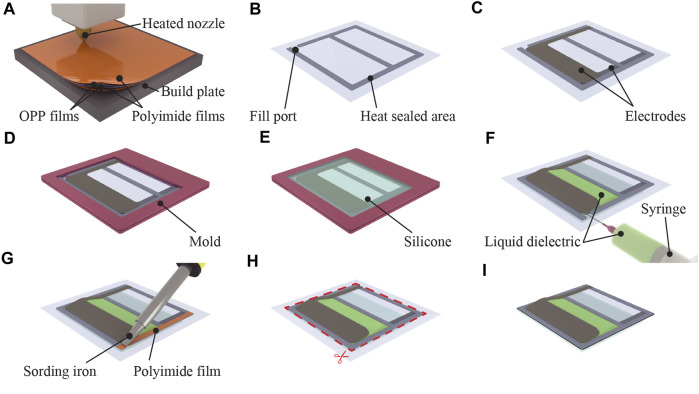
Fabrication process of the bending-type silicone-layered electrohydraulic soft actuators. **(A)** Heat sealing the OPP films using a 3D printer results in **(B)** a shell with a fill port. **(C)** Electrodes are attached on both sides of the shell. **(D)** Shell is placed in an acrylic mold. **(E)** Liquid silicone is poured into the mold, and the silicone is cured in an oven. **(F)** Shell is removed from the mold, and the liquid dielectric is injected. **(G)** Fill port is heat sealed with a soldering iron. **(H)** Unnecessary parts are removed to obtain **(I)** the finished actuator.

The fabrication process of the bending actuator is illustrated in Figure 2. The structure of the actuator fabricated via this process is shown in Figure 1A. Below, the fabrication process is explained according to each of the subfigures in Figures 2A–I. (a) The shell dimensions were set to 20 mm × 40 mm (length × width) with a seal width of 2.5 mm. A 3-mm margin without sealing was retained to facilitate the injection of the liquid dielectric. A 3D printer (Ultimaker 3 Extended, Ultimaker) was configured with a plate temperature of 50°C, head temperature of 200°C, and speed of 50 mm/s. Two OPP films were stacked with their sealing surfaces facing each other and enclosed between polyimide films. Subsequently, these were thermally sealed using the 3D printer, (b) generating a shell with a fill port. (c) The electrodes are then cut using a laser cutting machine (Speedy 300, Trotec) and attached to the lower halves of both sides of the shell. The electrode dimensions were chosen such that half of the shell, with a width of 40 mm and a length of 10 mm, was covered. (d) The shell with electrodes was inserted into an acrylic plate mold to form the silicone layer. The mold was designed and manufactured to ensure complete coverage of the electrodes with silicone, extending 2.5 mm beyond the left, right, and bottom edges of the electrodes. (e) The liquid silicone, which was prepared using the manufacturer’s recommended ratio (A part: B part = 1:1) was poured on one side. The mold filled with silicone was subsequently placed in an oven and cured at 50°C for 30 min. The thicknesses of the acrylic plates corresponded to the desired final silicone-layer thickness. The breakdown field of the silicone, i.e., Ecoflex 00-30, used in this study was 350 V/mil (Smooth-On, 2023). To withstand the test-voltage range of up to 10 kV, a minimum thickness of 0.73 mm was required. Therefore, by considering safety factors, the thickness of the silicone was set to 1 mm. (f) After removing the mold, 1 mL of the liquid dielectric was injected into the shell using a micropipette. (g) The injection port of the shell was sealed using a soldering iron set at 200°C to encapsulate the liquid dielectric. (h) The unnecessary parts were then trimmed. (i) The previously mentioned steps were used to fabricate the bending actuator. The linear actuator was fabricated using the same procedure, except for attaching the silicone layer on both sides of the actuator structure.

**FIGURE 3:**
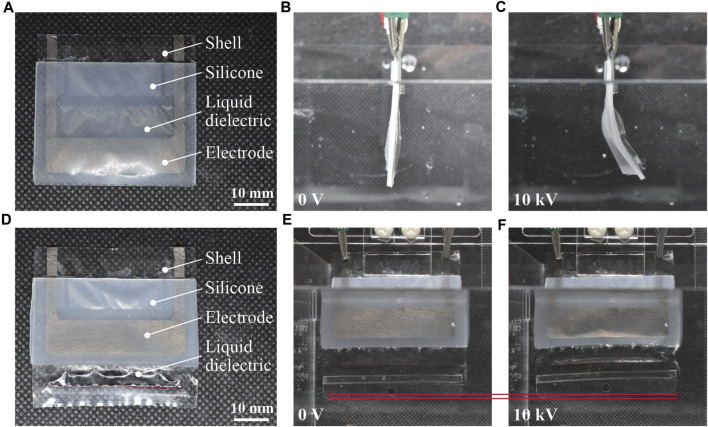
Fabricated silicone-layered electrohydraulic soft actuators and their movements. **(A)** Bending actuator immediately after fabrication, **(B)** initial state (zero voltage) in water, and **(C)** activated state with an applied voltage of 10 kV. **(D)** Linear actuator immediately after fabrication, **(E)** initial state (zero voltage) in water, and **(F)** activated state with an applied voltage of 10 kV.

**FIGURE 4:**
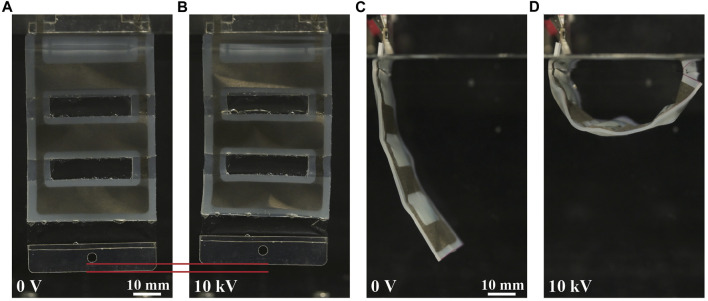
Movements of the three-shell underwater silicone-layered electrohydraulic soft actuators. **(A)** Three-shell linear actuator in the initial state (underwater, zero voltage) and **(B)** activated state with an applied voltage of 10 kV. **(C)** Three-shell bending actuator in the initial state (underwater, zero voltage) and **(D)** activated state with an applied voltage of 10 kV.

Figure 3 displays the fabricated actuators and their movements. As expected, we observed bending and linear actuators (contraction). As the actuators can be fabricated in a planar manner, the fabrication process allows them to have an increased number of shells. To validate this aspect, we fabricate and confirm the operation of bending and linear actuators with three shells, as shown in Figure 4. In these actuators, the individual shells were designed to match the dimensions (length × width) of a single actuator (20 mm × 40 mm), and a 2.5 mm gap was maintained between the shells.

### 2.3 Experimental methods

**FIGURE 5:**
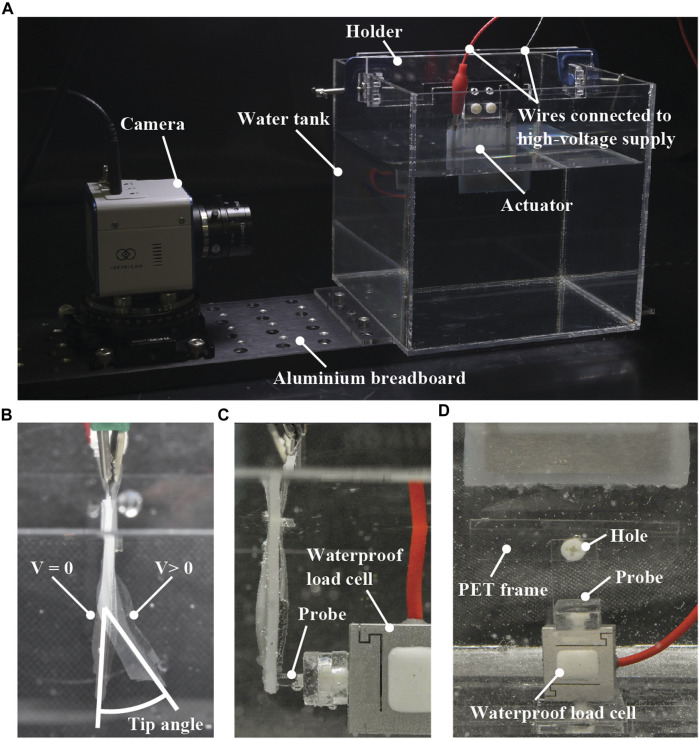
**(A)** A picture of the experimental setup used in this study. As the configuration of the setup shown this picture is for measuring the tip angle of the bending actuators, the load cell is absent. **(B)** Definition of the measured tip angle of the bending actuators. **(C)** Position of the load cell and its probe for measuring the blocked forces of the bending actuators. **(D)** PET sheet frame with a hole used for characterizing the linear actuators. This picture shows the case of measuring the blocked forces.

The fabricated actuators were characterized using a water tank filled with tap water. Figure 5A shows a picture of the experimental setup, where the water tank is fixed on an aluminum breadboard. On the water tank, an actuator sample is mounted using a holder made of an acrylic plate. Electric wires are connected to the terminals of the sample to apply the voltage from a high-voltage supply. The bottom of the water tank is flat so that a load cell for measuring blocked force can be placed with a jig made of an acrylic plate. Blocked force is the force generated at the tip of the actuator without any displacement. In addition, there is a mount on the breadboard that allows the fixing of different types of cameras.

The following characterization was performed using the experimental setup. The tip angle and blocked force of the single-shell bending actuators were measured. The contraction strain, blocked force, and frequency response of the linear actuators with a single shell were measured, followed by the calculation of power density. The cyclic responses of both bending and linear actuators with a single shell were also acquired. In addition to the characterization of the single-shell actuators, the tip angle and contraction strain of the three-shell actuators were measured from the bending and linear types, respectively. To clarify the influence of the silicone layer, the contraction strain of a single-shell linear actuator was measured in air for the cases with and without the silicone layer, and the power density was calculated.

#### 2.3.1 Measurement of tip angle, contraction strain, and blocked force

The tip angles of the bending actuators with a single shell and three shells were measured using a camera (Z5, Nikon), followed by image processing (ImageJ, NIH). The tip angle was defined as relative values between the initial (V = 0) and activated states (V > 0), as presented in Figure 5B. By using the same method, the contraction strains of the linear actuators with a single shell and three shells were measured under no load, a 5-g load, and a 10-g load. These loads were applied by hanging a weight from the hole on a 0.25-mm-thick polyethylene terephthalate (PET) sheet frame attached to the tip of the actuator (Figure 5D). This frame was attached using a silicone adhesive film (Polysil, Taiyo Wire Cloth). The contraction strain was defined as relative values between the initial (V = 0) and activated states (V > 0), as shown in Figures 3E,F and Figures 4A,B. The voltage applied was in the range of 0 V–10 kV in 1-kV increments. During these measurements, the voltage was applied to the actuator using a low-voltage power supply (PMX32-2QU, Kikusui Electronics) and a high-voltage DC/DC converter (CB101, XP Power).

The blocked forces of the bending and linear actuators with a single shell were measured using a waterproof load cell (LSb210, FUTEK). The equipment used for the tip angle and contraction strain measurements was also used to apply the voltage. The applied voltage was in the range of 0–10 kV in 1-kV increments. The blocked force was defined as a relative value between the initial (V = 0) and activated states (V > 0). The probe of the load cell was placed on the tip in the case of the bending actuator (Figure 5C) and fixed to the lower end in the case of the linear actuator (Figure 5D).

In the measurements described above, three samples were used for measuring the aforementioned parameters of each type of actuator, and the averages of the measured values were considered.

#### 2.3.2 Frequency response and cyclic responses

To investigate the frequency response, a 5-kV square wave was applied to a single-shell linear actuator to measure the contraction strains and blocked forces as a function of driving frequency. The contraction strain was measured at frequencies of 0.5, 1, 1.5, 2, 5, 10, and 15 Hz, whereas the blocked forces were measured at 1-Hz increments between 1 and 10 Hz. A high-speed camera (INFINICAM, Photron) at a frame rate of 100 fps was used to capture the measurements, followed by image processing. The blocked forces were measured using the waterproof load cell, and the average force of one cycle was calculated. The method for measuring the blocked forces was the same as that shown in Figure 5D. During the aforementioned measurements, a high-voltage DC/DC module (HOPP-10P(A), Matsusada) and a function generator (eK-FGJ, Matsusada) were used to drive the actuator.

To measure the cyclic response, a single-shell linear actuator and bending actuator each were turned on and off 1000 times with a frequency of 0.25 Hz. The applied voltage was set to 5 kV (square wave). The contraction strains of the linear actuator and the tip angle of the bending actuator at 0 V and 5 kV were measured at the 1st, 5th 10th, 50th, 100th, 500th, and 1000th cycles. The deformations were measured by processing the images captured by the camera. A high-speed camera (INFINICAM, Photron) was used during the measurement at a frame rate of 100 fps, followed by image processing. In this experiment, a previously reported polarity-reversal circuit (Mitchell et al., 2019) was used with a high-voltage DC/DC converter (CB101, XP Power) to ensure cyclic actuation (i.e., charging and discharging) of the actuator.

#### 2.3.3 Power density

To calculate the power density of the actuator, a 10-kV square wave was applied to the single-shell linear actuator with a 5-g load, and the displacement was captured using a high-speed camera (INFINICAM, Photron) at a frame rate of 100 fps. A high-voltage DC/DC module (HOPP-10P(A), Matsusada) and a function generator (eK-FGJ, Matsusada) were used to drive the actuator. The same experimental procedure was applied in air to a single-shell linear actuator with and without the silicone layer. In this case, water was removed from the water tank.

On the basis of the measured data, the velocity v was obtained from the displacement h and the frame rate f using the following equation:

v=hf. (1)

The work rate P is expressed as follows:

$$P=m_{w}gv=m_{w}ghf,$$ (2)

where $m_{w}$ is the mass of the applied load, g is the acceleration due to gravity, and v is the velocity of the actuator. By dividing the work rate P by the mass of the actuator $m_{a}$ , the power density U can be calculated using the following formula:

$$U=\frac{P}{m_{a}}=\frac{m_{w}ghf}{m_{a}}.$$ (3)

## 3 Results and discussion

### 3.1 Tip angle, contraction strain, and blocked force

**FIGURE 6:**
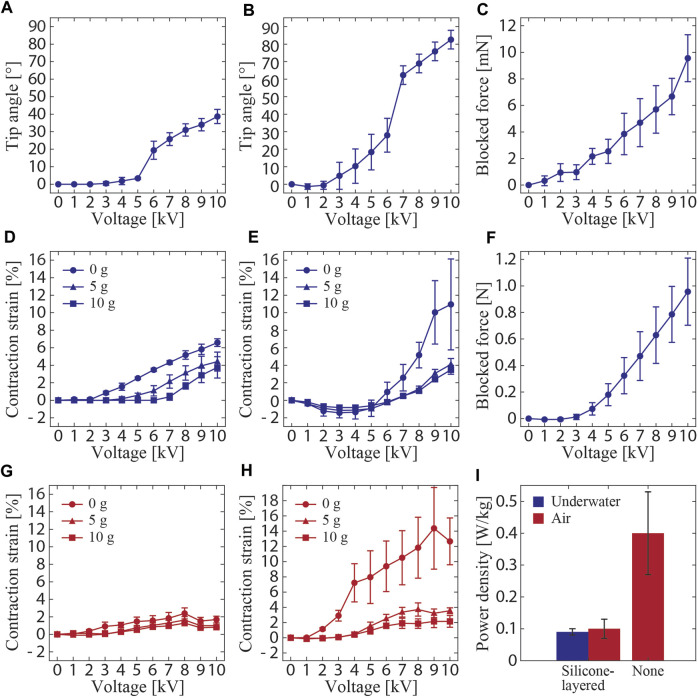
Measured performances of the silicone-layered electrohydraulic soft actuators as a function of applied voltage **(A–H)**. Tip angles of the **(A)** single-shell and **(B)** three-shell bending actuators. **(C)** Blocked force of the single-shell bending actuator. Contraction strains of the **(D)** single-shell and **(E)** three-shell linear actuators. **(F)** Blocked force of the single-shell linear actuator. **(G)** Contraction strain of the single-shell actuator measured in air. **(H)** Contraction strain of the single-shell actuator without the silicone layer measured in air. **(I)** Calculated power density of the single-shell actuator under different configurations and operating environments.

The results obtained from the characterizations revealed the voltage-controllable performance of the actuators. The tip angle of the bending actuator increased with the increase in voltage and reached a value of 39.0° at 10 kV (Figure 6A). The tip angle of the three-shell bending actuator was 82.6° at 10 kV (Figure 6B). Ideally, the tip angle of the three-shell actuator should be three times that of the single-shell actuator. However, the real tip angle of the three-shell actuator was approximately 2.1 times that of the single-shell one. This is because the deformation of the three-shell actuator includes pure bending and local deformation in multiple directions. In the measurement data, a plateau and a pull-in transition can be observed, which have been described as unique characteristics of such actuators (Acome et al., 2018). The pull-in transition occurred when the electrostatic force exceeded the restoring force threshold, resulting in a sudden increase in the amount of actuation. This means that most of the liquid dielectric between the electrodes is moved, followed by the plateau, where the amount of actuation is small with respect to the applied voltage. Furthermore, the blocked force of the bending actuator increased with increasing voltage and reached a value of 9.6 mN at 10 kV (Figure 6C). During the test, we observed that the tip of the actuator deformed and encased the probe of the load cell. This may have resulted in a bias in the deformation between the measured and the remaining areas, leading to a reduced measured force with respect to that of the linear actuator described later in this section.

The contraction strains of the linear actuator at 10 kV were measured as 6.6%, 4.4%, and 3.8% under the no load, 5-g load, and 10-g load conditions, respectively (Figure 6D). The strain decreased as the load increased. This behavior is consistent with the observations reported in other studies (Rothemund et al., 2021). A strain of 24%, which is larger than the value obtained in this study, was observed at 10 kV in a non-underwater actuator of the same type reported in the literature (Rothemund et al., 2021). This difference may be attributed to the presence of the silicone layer, which is a passive part hindering the electrostatic deformations between the electrodes, thus hindering actuation. The contraction strain of the three-shell linear actuator at 10 kV was measured as 11.0%, 4.1%, and 3.5% under no load, 5-g load, and 10-g load conditions, respectively (Figure 6E). The strain of the three-shell actuator under no load was significantly larger than that of the single-shell actuator. This may be attributed to the deformation of the actuator, including linear contraction and partial bending deformations; the apparent vertical displacement was found to be large. Therefore, the variance of data for this particular condition is larger than those for the actuator in other loading conditions. These partial bending deformations were suppressed when the actuator was loaded, which ensured that the device was vertical. Therefore, the contraction strains of the three-shell linear actuator under 5- and 10-g loads were almost equal to those of the single-shell actuator. Additionally, the blocked force of the single-shell linear actuator at 10 kV was 956.1 mN (Figure 6F), which was higher than that of the bending actuator, because the load cell was physically connected to the linear actuator during the measurement and the force was accurately transmitted.

The impact of the silicone layer is more visible for the contraction strains of the linear actuator in air. They were measured at 8 kV as 2.4%, 1.6%, and 1.3% under the no-load, 5-g load, and 10-g load conditions, respectively (Figure 6G). The weight of the silicone layer acts to inhibit actuation because the buoyancy caused by water is not working in this case. This assumption is further confirmed by the result obtained from the linear actuator without the silicone layer. In the voltage range of 8–9 kV, the actuator exhibited contraction strains of 14.4%, 3.7%, and 2.2% under no-load, 5-g load, and 10-g load conditions, respectively (Figure 6H). These values are clearly larger than those of the actuator with the silicone layer and are of the same order as those for a previously reported non-underwater actuator of the same type (Rothemund et al., 2021). The data obtained by the measurements in air (Figures 6G,H) showed the largest strains before the voltage reached the maximum tested value. This may be caused by the power required by the actuator exceeding the rated output in the air environment, where the effect of gravity is strong. The results discussed above suggest that, along with dielectric strength, the properties of the insulation layer (in this study, the silicone layer), such as volume, density, and modulus, are key parameters that define the performance of the waterproof electrohydraulic soft actuators.

### 3.2 Frequency and cyclic responses

The measured frequency responses of the contraction strains and blocked forces of the linear actuators are shown in Figures 6A,B. The strain decreased as the frequency increased. This indicated that the surrounding water dampened the actuation, as the hydrodynamic drag depends on the velocity. The same should be applicable to the bending actuators. In this case, the area subjected to drag forces during the actuation is larger, so the influence of the hydrodynamic drag is also expected to be larger. Therefore, it is suggested that the behavior of the surrounding fluid, including drag forces, should be considered as a limiting factor for the actuation of the underwater actuators in this study. Regarding the blocked force, it reached a maximum at approximately 3 Hz, and the force was higher than that when a DC (0 Hz) voltage was applied. This may be because of the resonance mode induced by the rigidity of the system, including that of the load cell.

**FIGURE 7:**
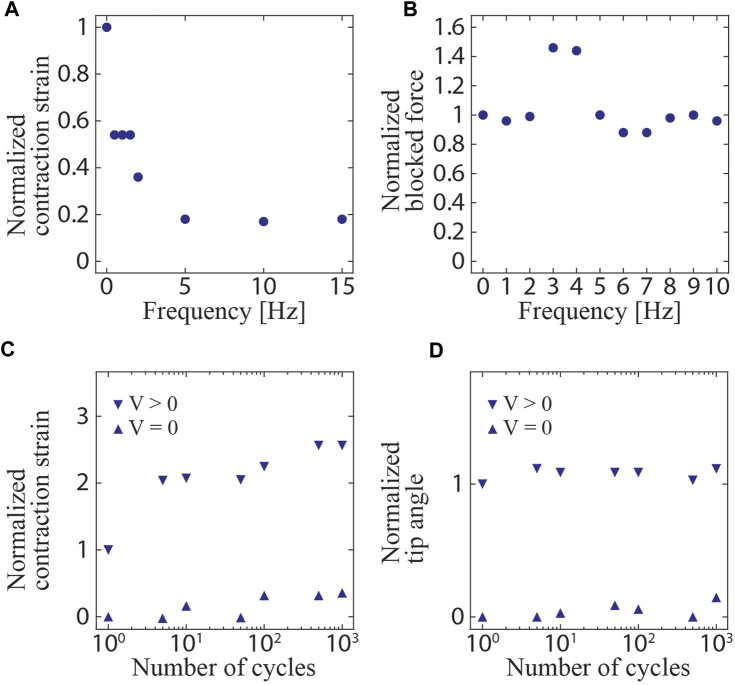
Measured frequency and cyclic responses. **(A)** Contraction strain and **(B)** blocked force of the linear actuator as a function of driving frequency (normalized at 0 Hz). **(C)** Contraction strain of the linear actuator as a function of the number of actuation cycles (normalized at the first actuated strain). **(D)** Tip angle of the bending actuator as a function of the number of actuation cycles (normalized at the first actuated angle).

The measured cyclic responses of the linear and bending actuators are presented in Figure 7C and Figure 7D, respectively. The actuators withstood 1000 on/off cycles and showed only a slight change in the strain and tip angle over the tested cycles. Remarkably, the linear actuator exhibited a low strain at the first actuation. This may have resulted from an error in the experimental setup causing a short activation time to charge, leading to a low strain. Nevertheless, the results indicate the high durability of the actuators.

### 3.3 Power density

Based on the measured data and Eq. 1, the actuation velocity v of the linear actuator was obtained as 8.6 × 10^−3^ m/s. From the mass of the load ( $m_{w}$ = 5.0 × 10^−3^ kg) and the acceleration due to gravity ( g = 9.8 m/s^2^), the work rate P was calculated as 4.23 × 10^−4^ W using Eq. 2. The mass of the actuator was measured to be 3.87 g. The power density U of the single-shell linear actuator was subsequently calculated using Eq. 3 as 0.09 W/kg, which was lower than that of the non-waterproof actuator of the same type (50–160 W/kg) (Kellaris et al., 2018). Two factors were mainly considered with regard to the low power density. The first was the presence of the surrounding water that resisted and decelerated the movement of the actuator. The second was the silicone layer that contributed to the decrease in power density, with its mass accounting for 2/3 of the total mass of the actuator. As the silicone layer is passive, the input energy is used for its deformation, thereby reducing the power density. These assumptions can be confirmed further by the power density of the single-shell linear actuator with (0.10 W/kg) and without the silicone layer (0.40 W/kg) in air, as presented in Figure 6I.

## 4 Bio-inspired underwater robots and soft robotics applications

To demonstrate the applicability of the developed actuators toward bio-inspired underwater robots, the bending and linear actuators were incorporated into ray and fish robots, respectively. Moreover, to investigate the versatility of the actuators for use in soft robotics systems, a water-surface sliding robot and an underwater gripper were fabricated based on the bending actuator. In these devices, the connections between the electrical wires and electrodes were covered with a molded 2-mm thick silicone layer to ensure waterproofing. Moreover, in the robots except for the gripper, the electrical wires were connected to 50-µm-diameter enameled wires in the middle to reduce the mechanical resistance while the robots are in motion.

### 4.1 Ray robot

The structure and swimming motion of the ray robot are illustrated in Figures 7A,B. The robot consisted of two bending actuators attached to a body frame constructed using styrene foam. Each actuator had a fin composed of an OPP film and a 0.25-mm-thick PET sheet frame. Every material was processed by a laser cutting machine and attached using double-sided tape. The total length of the robot was 50 mm. When a voltage was simultaneously applied to the two actuators, their bending deformations were converted to a flapping motion of the PET frame that caused the OPP film to passively deform. The OPP film in the fin produced a net thrust force in the horizontal direction, i.e., along the water surface, leading to a swimming motion. When a square wave of 9 kV at 5 Hz was applied to the robot, a swimming speed of 10.5 mm/s (0.21 body length/s (BL/s)) was achieved. The movement of the robot is shown in Figure 9A and Supplementary Video S1.

### 4.2 Fish robot

**FIGURE 8:**
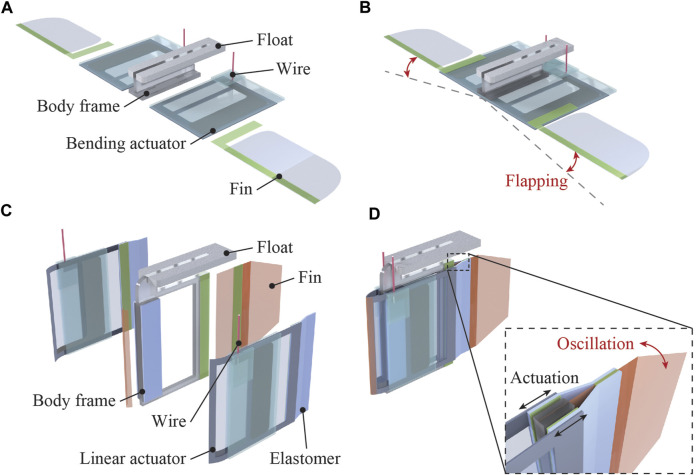
Structure and swimming motion of the bio-inspired robots using silicone-layered electrohydraulic soft actuators. **(A)** Structure of the ray robot employing two bending actuators. **(B)** Cyclic activation of the actuators yields a flapping swimming motion, generating a thrust force. **(C)** Structure of the fish robot with two linear actuators placed antagonistically. **(D)** Oscillating swimming motion of the tail fin is realized when the two actuators are activated individually, generating a thrust force propelling the robot forward.

**FIGURE 9:**
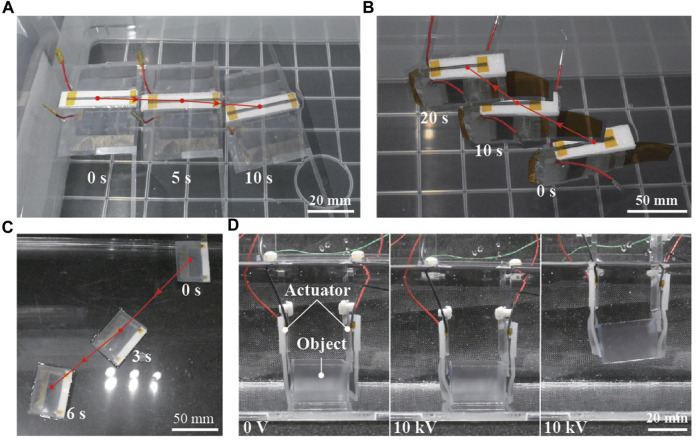
Motion sequences of the fabricated bio-inspired underwater robots and soft robotics applications. **(A)** Ray robot, **(B)** fish robot, **(C)** water-surface sliding robot, and **(D)** underwater gripper.

The fish robot consists of two linear actuators placed in an antagonistic configuration, as represented in Figure 8. One end of the actuator was attached to a tail fin made of a 50-µm-thick polyimide film via a pre-stretched elastomer membrane (VHB4905, 3M). The other end of the actuator and the hinge mechanism were attached to the body and constructed using a 2-mm-thick acrylic plate. To reduce drag in the water, a polyimide tape with an acute angle was placed at the head. The total length of the robot was 80 mm. When a voltage was applied to one of the actuators, the tail fin bent toward the activated side. By alternately applying voltages to the two actuators, the tail fin oscillated to the left and right, generating a propulsive force. Figure 9B shows the swimming motion of the robot (refer to Supplementary Video S1). A square wave of 8 kV with 5 Hz frequency was alternately applied to the two actuators, resulting in a swimming speed of 6.0 mm/s (0.07 BL/s).

### 4.3 Soft robotic applications

By attaching a float made of 3-mm-thick styrene foam to a bending actuator, a simple robot that slides on the water surface was constructed. When a voltage was applied, the tip of the actuator moved up and down, pushing the water to the rear and moving forward. The total length of the robot was 35 mm. A swimming speed of 31.2 mm/s (0.91 BL/s) was achieved when a square wave of 8 kV at 0.5 Hz frequency was applied to the robot (Figure 9C) (refer to Supplementary Video S1). The locomotion speed of the robot was considerably higher than that of the other robots discussed above. This may be attributed to the lower drag force acting on the sliding robot than those acting on the others because this robot was floating on the water’s surface.

The bending actuator also demonstrated its ability to conduct underwater grasping tasks. A gripper was fabricated by integrating two bending actuators via a fixed part made of acrylic plates. As observed in Figure 9D and Supplementary Video S1, the gripper could retrieve and place a 3D-printed box in a water environment by controlling the on and off modes of the applied voltage (10 kV).

## 5 Conclusion

In this study, we developed silicone-layered waterproof electrohydraulic soft actuators for constructing bio-inspired underwater robots. Two types of actuators, namely, bending and linear actuators were fabricated and characterized. The experimental results revealed voltage-controllable performances of the actuators in a water environment; therefore the implementation of the concept was successful. Based on the developed actuators, several aquatic robots with different morphologies and swimming modes, as well as a gripper, were fabricated, which exhibited underwater operations as expected. Therefore, the actuators demonstrated high potential for bio-inspired underwater robots and soft robotics applications.

Future work on this topic will include improvements in the actuation performance and investigation of the robot morphologies and swimming modes that can utilize the actuators. Although the use of silicone as an insulation material enabled the actuators to operate underwater, their performances were relatively lower than those of the non-waterproof types because the silicone layers acted as passive components. To address this issue, the insulation material can be changed to a material with higher breakdown voltage and lower Young’s modulus than those of silicone. A material with these features will enable insulation with thin and compliant nature, minimizing mechanical resistance and improving actuator performance. Optimizing the dimensions of the electrodes, flexible shell, and the amount of liquid dielectric is another possible approach to increase actuation performance. An analytical model and a simulation environment can be used to optimize actuator performance. Here, the incorporation of hydrodynamics and consideration of the properties and conditions of the surrounding fluid are important aspects to consider. Hydrodynamics should also be considered in the experimental conditions for characterizing real actuators and robots to further elucidate their abilities. These research initiatives will lead to the realization of underwater robots with various morphologies and soft robotics applications.

## Data Availability

The raw data supporting the conclusion of this article will be made available by the authors, without undue reservation.

## References

[B1] Acome E. Mitchell S. K. Morrissey T. G. Emmett M. B. Benjamin C. King M. (2018). Hydraulically amplified self-healing electrostatic actuators with muscle-like performance. Science 359, 61–65. 10.1126/science.aao6139 29302008

[B2] Aracri S. Giorgio-Serchi F. Suaria G. Sayed M. E. Nemitz M. P. Mahon S. (2021). Soft robots for ocean exploration and offshore operations: a perspective. Soft Robot. 8, 625–639. 10.1089/soro.2020.0011 33450174 PMC8713554

[B3] Aureli M. Kopman V. Porfiri M. (2010). Free-locomotion of underwater vehicles actuated by ionic polymer metal composites. IEEE/ASME Trans. Mechatronics 15, 603–614. 10.1109/TMECH.2009.2030887

[B4] Coral W. Rossi C. Curet O. M. Castro D. (2018). Design and assessment of a flexible fish robot actuated by shape memory alloys. Bioinspir. Biomim. 13, 056009. 10.1088/1748-3190/AAD0AE 29968572

[B5] Jian X. Zou T. (2022). A review of locomotion, control, and implementation of robot fish. J. Intell. Robot. Syst. 106, 37. 10.1007/s10846-022-01726-w

[B6] Katzschmann R. K. DelPreto J. MacCurdy R. Rus D. (2018). Exploration of underwater life with an acoustically controlled soft robotic fish. Sci. Robot. 3, eaar3449. 10.1126/scirobotics.aar3449 33141748

[B7] Kellaris N. Gopaluni Venkata V. Smith G. M. Mitchell S. K. Keplinger C. (2018). Peano-HASEL actuators: muscle-mimetic, electrohydraulic transducers that linearly contract on activation. Sci. Robot. 3, eaar3276–11. 10.1126/scirobotics.aar3276 33141696

[B8] Li G. Chen X. Zhou F. Liang Y. Xiao Y. Cao X. (2021). Self-powered soft robot in the mariana trench. Nature 591, 66–71. 10.1038/s41586-020-03153-z 33658693

[B9] Li G. Liu G. Leng D. Fang X. Li G. Wang W. (2023). Underwater undulating propulsion biomimetic robots: a review. Biomimetics 8, 318. 10.3390/biomimetics8030318 37504206 PMC10807579

[B10] Mitchell S. K. Wang X. Acome E. Martin T. Ly K. Kellaris N. (2019). An easy‐to‐implement toolkit to create versatile and high‐performance HASEL actuators for untethered soft robots. Adv. Sci. 6, 1900178. 10.1002/advs.201900178 PMC6662077 31380206

[B11] Nguyen D. Q. Ho V. A. (2022). Anguilliform swimming performance of an eel-inspired soft robot. Soft Robot. 9, 425–439. 10.1089/soro.2020.0093 34134542

[B12] Rothemund P. Kellaris N. Mitchell S. K. Acome E. Keplinger C. (2021). HASEL artificial muscles for a new generation of lifelike robots—recent progress and future opportunities. Adv. Mater 33, 2003375. 10.1002/adma.202003375 PMC11469257 33166000

[B13] Shintake J. Cacucciolo V. Shea H. Floreano D. (2018). Soft biomimetic fish robot made of dielectric elastomer actuators. Soft Robot. 5, 466–474. 10.1089/soro.2017.0062 29957131 PMC6101101

[B14] Shintake J. Ming A. Shimojo M. (2010). “Development of flexible underwater robots with caudal fin propulsion,” in 2010 IEEE/RSJ International Conference on Intelligent robots and Systems, Taipei, Taiwan, 18-22 October 2010 (IEEE), 940–945.

[B15] Smooth-On (2023). EcoflexTM Series. Available at: https://www.smooth-on.com/tb/files/ECOFLEX_SERIES_TB.pdf (Accessed September 22, 2023).

[B16] Takagi K. Yamamura M. Luo Z. Onishi M. Hirano S. Asaka K. (2006). “Development of a rajiform swimming robot using ionic polymer artificial muscles,” in 2006 IEEE/RSJ International Conference on Intelligent Robots and Systems (IEEE), Beijing, China, 09-15 October 2006 (IEEE), 1861–1866.

[B17] Villanueva A. Smith C. Priya S. (2011). A biomimetic robotic jellyfish (Robojelly) actuated by shape memory alloy composite actuators. Bioinspir. Biomim. 6, 036004. 10.1088/1748-3182/6/3/036004 21852714

[B18] Wang R. Zhang C. Zhang Y. Tan W. Chen W. Liu L. (2023a). Soft underwater swimming robots based on artificial muscle. Adv. Mater. Technol. 8, 2200962. 10.1002/admt.202200962

[B19] Wang T. Joo H. J. Song S. Hu W. Keplinger C. Sitti M. (2023b). A versatile jellyfish-like robotic platform for effective underwater propulsion and manipulation. Sci. Adv. 9, eadg0292. 10.1126/sciadv.adg0292 37043565 PMC10096580

[B20] Xing J. Jin W. Yang K. Howard I. (2023). A bionic piezoelectric robotic jellyfish with a large deformation flexure hinge. IEEE Trans. Ind. Electron. 70, 12596–12605. 10.1109/TIE.2023.3234155

